# Association between metal cobalt exposure and the risk of congenital heart defect occurrence in offspring: a multi-hospital case-control study

**DOI:** 10.1186/s12199-020-00877-2

**Published:** 2020-08-08

**Authors:** Nannan Zhang, Shuihua Yang, Jiaxiang Yang, Ying Deng, Shengli Li, Nana Li, Xinlin Chen, Ping Yu, Zhen Liu, Jun Zhu

**Affiliations:** 1 National Center for Birth Defect Monitoring, Key Laboratory of Birth Defects and Related Diseases of Women and Children, Ministry of Education, West China Second University Hospital, and State Key Laboratory of Biotherapy, Sichuan University, Sec. 3 No.17, South Ren Min Road, Chengdu, 610041 Sichuan People’s Republic of China; 2 Department of Ultrasound, Maternal and Child Healthcare Hospital of Guangxi Zhuang Autonomous Region, Nanning, Guangxi People’s Republic of China; 3 Department of Ultrasound, Sichuan Maternal and Child Healthcare Hospital, Chengdu, 610041 Sichuan People’s Republic of China; 4 Department of Ultrasound, Shenzhen Maternity and Child Healthcare Hospital, Shenzhen, Guangdong People’s Republic of China; 5 Department of Ultrasound, Hubei Maternal and Child Healthcare Hospital, Wuhan, Hubei People’s Republic of China

**Keywords:** Cobalt, Metal exposure, Congenital heart defect, Hair biomarker, Placenta tissue

## Abstract

**Background:**

Many studies have investigated heavy metal exposure could increase the occurrence of congenital heart defects (CHDs). However, there are limited data regarding the relationship between cobalt exposure and CHD occurrence in offspring. The aim of this study was to analyze the association between cobalt exposure in mothers and the risk of CHDs in offspring.

**Materials and methods:**

In order to explore the association between cobalt exposure and occurrence of congenital heart defect (CHD), a case-control study with 490 controls and 399 cases with CHDs in China were developed. The concentrations of cobalt in hair of pregnant woman and fetal placental tissue were measured and processed by a logistic regression analysis to explore the relationship between cobalt exposure and risk of CHDs.

**Results:**

The median concentration of hair cobalt in the control and case group was 0.023 ng/mg and 0.033 ng/mg (aOR, 1.837; 95% CI, 1.468–2.299; *P* < 0.001), respectively. And the median (5–95% range) fetal placental cobalt concentrations were 19.350 ng/g and 42.500 ng/g (aOR, 2.924; 95% CI, 2.211–3.868; *P* < 0.001) in the control and case groups, respectively. Significant differences in the middle level of cobalt in hair were found in the different CHD subtypes, including septal defects, conotruncal defects, right ventricular outflow tract obstruction, and left ventricular outflow tract obstruction (*P* < 0.001). Dramatically, different cobalt concentrations in fetal placental tissue were found in all subtypes of cases with CHDs (*P* < 0.01).

**Conclusions:**

The finding suggested that the occurrence of CHDs may be associated with cobalt exposure.

## Introduction

Congenital heart defects (CHDs) have an incidence of 6–8 per 1000 at birth [1] and are the leading cause of perinatal and infant death [2]. Patients with CHDs require long-term specialty care according to the severity of the defect [3, 4]. Epidemiologic studies showed that patients with CHD have an accompanying great economic burden for families and society [5]. CHDs, a multifactorial complex disease, could be caused by genetic factors and environmental factors [6, 7]. Environmental factors were one of the most important pathogenic factors of CHD, such as exposure to hypoxia, nitrogen dioxide, dilantin, halogenated hydrocarbons, and retinoic acid, increasing the risk of CHD [8–10].

Many studies have shown that exposure to metals, such as lead, barium, cadmium, arsenic, and copper, also increases the risk of CHDs [11, 12]. Cobalt (Co), a relatively rare element in the Earth’s crust, is essential to mammals in the form of cobalamin (vitamin B12) [13]. Cobalt metal, salts, hard metals, oxides, and alloys are the most commercially important cobalt compounds [14]. Cobalt can be found in the air, seawater, forest fire, volcanic eruptions, and erosion [15]. Anthropogenic use is also the main source of cobalt exposure, such as fossil fuel, engine emissions, Co-containing alloys, diamond polishing, and steel [15]. Diet, inhalation air, and drinking water are also the main source of cobalt in the general population [16]. The highest levels of Co are found in chocolate, green leafy vegetables, fresh cereals, offa, coffee, fish, and nuts [17]. Cobalt may originate from environmental sources, such as food processing and packaging, industrial, or other anthropogenic activities [18]. High concentrations of cobalt exposure may be observed in certain occupations, such as hard metal alloy processing and prosthesis use [19]. Inhalation of cobalt dust represents the main source of exposure in occupational workers [20]. Therefore, the sources and pathways of human cobalt exposure are diverse.

Cobalt plays an important role in nucleic acid synthesis, amino acid synthesis, erythrocyte formation, and vitamin B12 formation [21]. Cobalt accumulates primarily in the heart, pancreas, liver, and kidney, with the relative content in skeletal muscle and the skeleton increasing with time after cobalt uptake [22]. However, cobalt exposure could cause allergic contact dermatitis, asthma, lung fibrosis, hepatotoxicity, and caners [19, 23, 24]. Exposure to cobalt also leads to nervous system injury, for example memory loss, neuropathies, optic atrophy, and bilateral nerve deafness [15, 25]. Cobalt metal and salts (mainly cobalt chloride and cobalt sulfate) have genotoxicity, including micronuclei or DNA damage and chromosomal aberrations, probably due to oxidative stress [26]. Additionally, cobalt exposure could induce adult cardiovascular diseases. Animal studies showed that exposure to 2.0–7.6 mg Co/kg-day led to cardiovascular toxic effects [21]. Worker exposure to cobalt (0.1–5 mg Co/m^3^) had a higher risk of cardiovascular diseases, including dilative cardiomyopathy, higher heart rate, coronary heart disease, hypertension, and valvular heart disease [27–29]. Animal studies also showed that mice consuming cobalt in the drinking water led to reproductive toxicity, including a reduction in fertility, a decrease in the number of implantation sites and viable fetuses, and a loss of testicular weight [30]. Above all, high levels of cobalt exposure have been a serious threat to environmental safety and public health.

However, research has paid little attention to the association between cobalt levels and the risk of congenital heart defects. The purpose of this study was to examine the interaction between cobalt exposure and CHDs. In this study, we collected clinical data and analyzed the cobalt concentration in pregnant mothers’ hair and fetus’ placenta tissues to investigate the association between maternal cobalt exposure and the risk of occurrence of CHDs in offspring.

## Materials and methods

### Study population

The case-control study was conducted from August 2010 to July 2013 at five maternal and child hospital in the cities of Fuzhou, Zhengzhou, Shenzhen, Wuhan and Xi’an, China. Fetuses diagnosed with cardiac defects prenatally were selected as the cases. Pregnant woman who had healthy fetus was selected in the same hospital with gestation ages within 2 weeks differ from the case fetus. All live cases and controls were examined by pediatric cardiologists within the first week after clinical diagnosis by heart auscultation and neonatal echocardiography. Terminated pregnancies or stillbirths were established according to autopsy reports. Then, all clinical data and samples were collected after recruitment. More details regarding the recruitment procedure were provided in our previous study [11]. Each participant was informed during the enrolment process. The study was approved by the Ethics Committee of Sichuan University (No. 2010004).

The gestational ages of all subjects ranged from 14 to 40 weeks, and exclusion criteria for cases and controls included (1) mothers unwilling to participate or with mental disease or dyed hair, (2) fetuses with chromosomal abnormalities or hereditary syndrome or unclear diagnosis, (3) multi-fetal pregnancy, and (4) fetuses with CHD family history. All CHD cases were divided into six major categories according to the anatomic lesion as described in a previous study [12]: (a) septal, (b) conotruncal, (c) right-sided obstructive, (d) left-sided obstructive, (e) anomalous venous return, and (f) other cardiac structural abnormalities.

### Questionnaire interview and sample collection

All participants recruited in the study received a face-to-face interview during the antenatal examination. The questionnaire information was obtained as follows: pregnancy history, working and living environment, life style, maternal diet and nutrition, drug use history, family history, maternal illness, and folic acid supplementation. Information regarding potential confounders was obtained for covariate analysis.

Maternal hairs weighing 0.1 g and 3 to 5 cm long were collected from the occipital area after the interview. Placental tissues of approximately 1 cm^3^ were sheared from the fetal surface of the placenta after delivery. All samples were kept in individual labeled sterile microtubes and frozen at − 80 °C until use. More details of sample collection were provided in previous studies [31].

### Concentration analysis of cobalt in human tissues

Human hair standard reference materials (GBW09101) were obtained from the Shanghai Institute of Nuclear Research. The cobalt concentrations of the samples were analyzed as described previously using an Agilent 7500cx ICP/MS system (Agilent Technologies; Wilmington, DE) equipped with a G3160b I-AS integrated autosampler [32]. Metals including iron (Fe) and calcium (Ca) were measured together with cobalt from same hairs of these study participants. The limit of detection for cobalt in hair was 0.01 ng/mg, and that for cobalt in placenta tissue was 0.40 ng/g and the limit detected concentration of iron was 0.01 ng/mg in hair and 0.18 ng/g in placenta tissues, respectively. The detection limit for calcium was 0.1 ng/mg in hair and 0.89 ng/g in placenta tissues, respectively.

### Statistical analysis

Data analysis was performed using the SPSS 17.0 software (Chicago, IL, USA). A case-control analysis was performed to assess the potential effects of cobalt using data from identified cases and controls. Differences in demographic information and maternal characteristics between the control and case groups were compared by chi-square tests (two-tailed values of *P* < 0.05). The distributions of cobalt concentration were analyzed by one-sample Kolmogorov-Smirnov tests. The distributions of cobalt levels are presented as medians, arithmetic means, and 5–95% ranges. Differences in cobalt levels between the case and control groups were assessed by Wilcoxon-Mann-Whitney tests.

The association between the risks of CHDs and cobalt exposure was assessed by crude odds ratios (cORs), adjusted odds ratios (aORs), and 95% confidence intervals (95% CIs) using logistic regression. The potential confounding effects were maternal age, gestational age, body mass index (BMI), education, parental smoking habits, folic acid supplementation, and iron and calcium concentration.

The transformed cobalt concentration was normalized using the Napierian logarithm and divided into tertiles (low, medium, and high), and the first tertile of cobalt (hair cobalt: ≤ 0.0210 ng/mg, fetal placental cobalt: ≤ 19.8281 ng/g) was considered the reference. Two-tailed *P* values < 0.05 and 95% CIs excluding 1.00 were considered statistically significant.

## Results

### Characteristics of participants

During the study period, all 1776 mothers were recruited. According to the exclusion criteria, 568 cases were excluded due to extracardiac abnormalities in case group or any malformations detected after births in control group. Additionally, 228 mothers were excluded due to loss of collection of specimens. The total number of hair samples was 595. The number of hair samples in the case group and control group was 269 and 326, respectively. The total number of placental tissue samples was 393, and the number of placental tissue samples in the case group and control group was 181 and 212, respectively. The maternal characteristics of the samples are listed in Table 1. Gestational age, folic acid supplementation, parental smoking, and education level of the mother were significantly different between the two groups (*P* < 0.05), while there were no significant differences in maternal age (*P* = 0.099) and BMI (*P* = 0.136).

**Table 1:** Descriptive characteristics of the study sample

Variable	Control, *n* = 490 (%)	Cases, *n*= 399 (%)	Chi square	*P* value
Maternal age (years, *n*) ^a^			7.803	0.099
*n* < 20	12 (2.4)	12 (3.0)		
20 ≤ *n* < 25	111 (22.7)	113 (28.3)		
25 ≤ *n* < 30	216 (44.1)	179 (44.9)		
30 ≤ *n*< 35	108 (22.0)	73 (18.3)		
*n* ≥ 35	43 (8.8)	22 (5.5)		
Gestational age (week, *n*) ^a^			37.628	< 0.001***
*n* < 15	6 (1.2)	2 (0.5)		
15 ≤ *n*< 20	69 (14.1)	15 (3.8)		
20 ≤ *n* < 25	233 (47.6)	182 (45.6)		
26 ≤ *n*<31	109 (22.2)	136 (34.1)		
*n* ≥ 31	73 (14.9)	64 (16.0)		
Folic acid supplement			11.350	0.001**
Yes	436 (89.0)	323 (81.0)		
No	54 (11.0)	76 (19.0)		
Parental smoking			15.615	*<* 0.001***
Yes	181 (36.9)	200 (50.1)		
No	309 (63.1)	199 (49.9)		
ppBMI (kg/cm^2^)			3.987	0.136
BMI < 18.5	120 (23.1)	116 (27.6)		
18.5 ≤ BMI< 25	381 (73.3)	295 (70.2)		
BMI ≥ 25	19 (3.7)	9 (2.1)		
mEDU			38.113	*<* 0.001***
Primary school and below	3 (0.6)	16 (4.0)		
Junior middle school	77 (15.7)	114 (28.6)		
Senior high school	124 (25.3)	89 (22.3)		
College degree and above	284 (58.0)	173 (43.4)		

^*a*^Using base data in following multivariate analysis as continuous variables

**P < 0.01, ***P < 0.001, two-tailed test, there was statistically significant difference between groups

### Cobalt concentration in hair samples

**Fig. 1:**
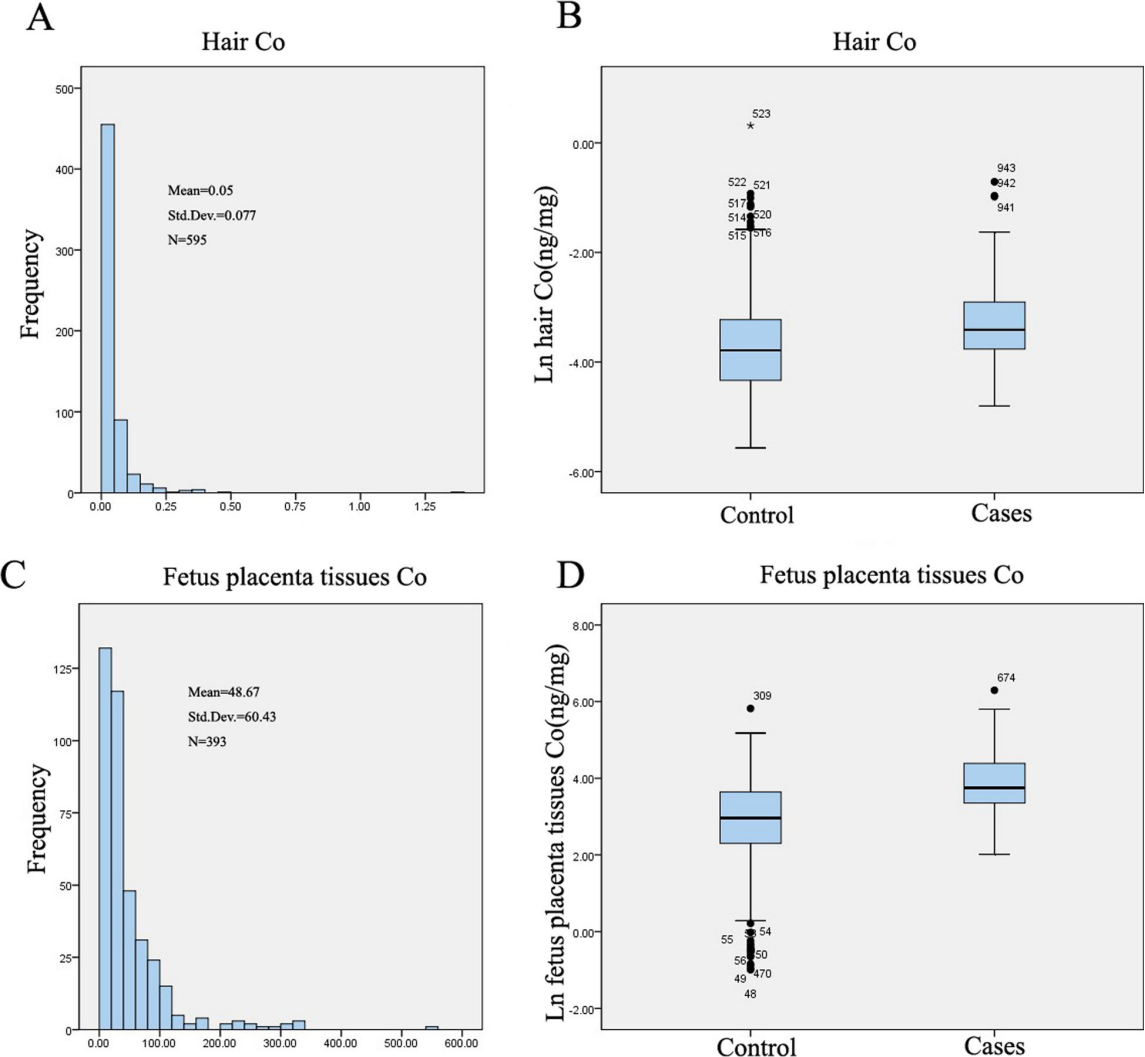
Levels and frequency of cobalt in CHDs and control groups. **a** Frequency of cobalt in maternal hair. **b** Boxplots of cobalt levels (Napierian logarithm transformed) of hair samples. **c** Frequency of cobalt in fetus placental tissues. **d** Boxplots of cobalt levels (Napierian logarithm transformed) in fetus placental tissues. The line inside the box = medians; the box length = interquartile range (IQR); the upper and lower ends = 95 and 5% value. One-sample Kolmogorov-Smirnov test was used to verify the distributions of cobalt. The distributions of cobalt did not conform to normal distribution. ***P < 0.001, cases compared to the control group

**Fig. 2:**
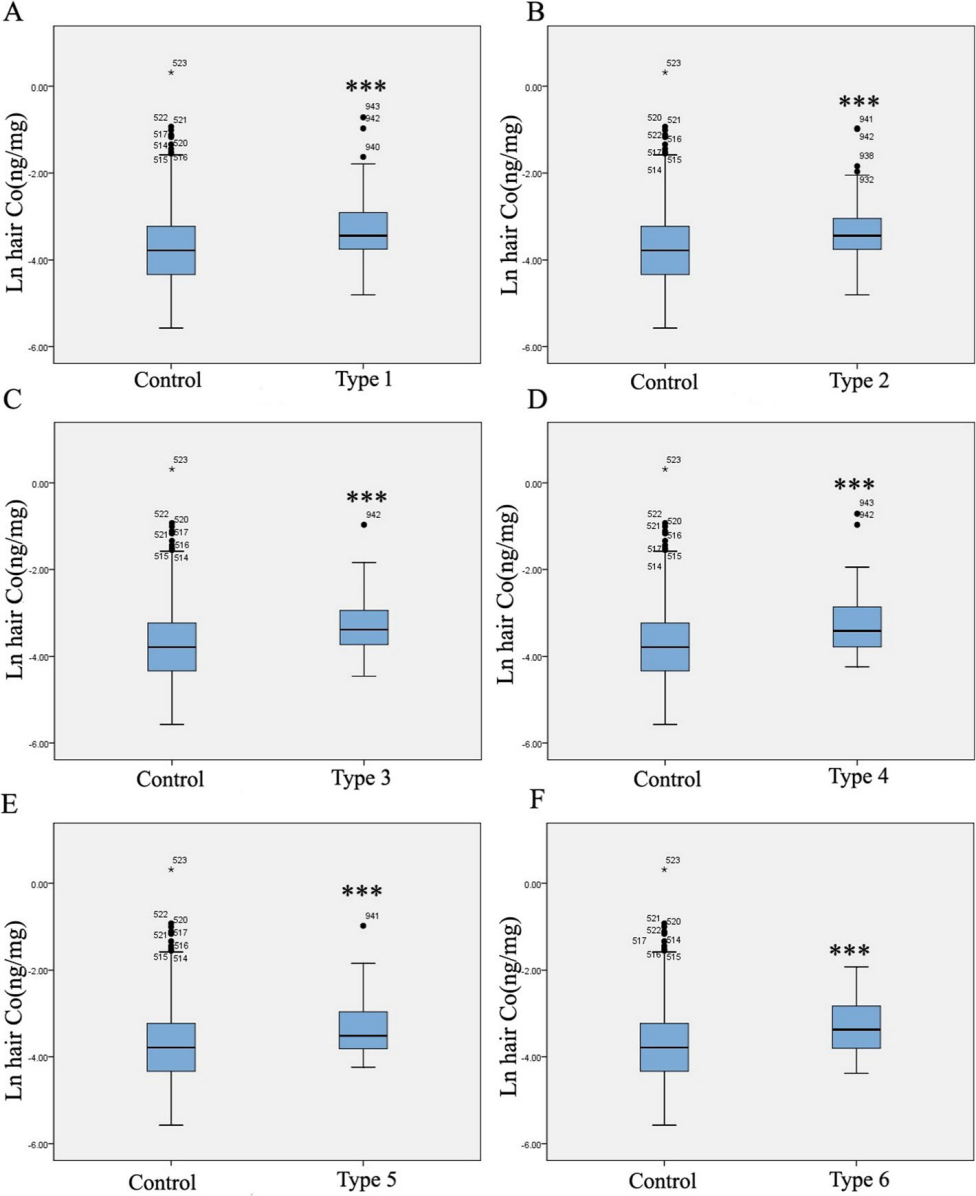
Levels of hair cobalt in CHD subtypes and control groups. Boxplots of cobalt levels (Napierian logarithm transformed) of hair samples. The line inside the box = medians; the box length = interquartile range (IQR); the upper and lower ends = 95 and 5% value. One-sample Kolmogorov-Smirnov test was used to verify the distributions of cobalt. ***P < 0.001, cases compared to the control group. Type 1, septal defect; type 2, conotruncal defects; type 3, right ventricular outflow tract obstruction; type 4, left ventricular outflow tract obstruction; type 5, anomalous pulmonary venous return; type 6, other heart defects

Levels of metal hair cobalt were compared between the control and case groups. The median concentrations (5–95% range) of hair cobalt in the control and case groups were 0.023 ng/mg (0.007–1.174 ng/mg) and 0.033 ng/mg (0.016–0.134 ng/mg), respectively (Table 2). The hair cobalt concentrations in subtypes of CHDs are presented in Table 2. There were significant differences (*P* < 0.001) between each CHD subtype for cases and controls. After the value was Napierian logarithm transformed, the concentrations of hair cobalt in the CHD group were significantly higher than those in the control group (*P* < 0.001) (Figs. 1 and 2).

**Table 2:** Descriptive statistics for hair cobalt level in the case and control groups

	Hair cobalt (ng/mg)							
	*n*	AM	5th p	25th p	Median	75th p	95th p	*P* value
Control	326	0.045	0.007	0.013	0.023	0.040	0.174	
All case	269	0.047	0.016	0.023	0.033	0.055	0.134	< 0.001***
Case with septal defects	183	0.047	0.016	0.023	0.0318	0.054	0.140	< 0.001***
Case with conotruncal defects	133	0.045	0.016	0.023	0.032	0.048	0.117	< 0.001***
Case with right ventricular outflow tract obstruction	123	0.045	0.017	0.027	0.034	0.054	0.122	< 0.001***
Case with left ventricular outflow tract obstruction	42	0.051	0.015	0.022	0.030	0.052	0.154	< 0.001***
Case with anomalous pulmonary venous return	42	0.086	0.013	0.025	0.032	0.099	0.326	0.001***
Other heart defects	48	0.045	0.014	0.022	0.034	0.060	0.115	0.001***

*n* number; *AM* arithmetic means; *5th p*, *95th p* lead level in 5%, 95% percentiles respectively

***P < 0.001, two-tailed test, Wilcoxon-Mann-Whitney on nonparametric test compared to the control group

### Cobalt levels in fetal placental tissues

**Fig. 3:**
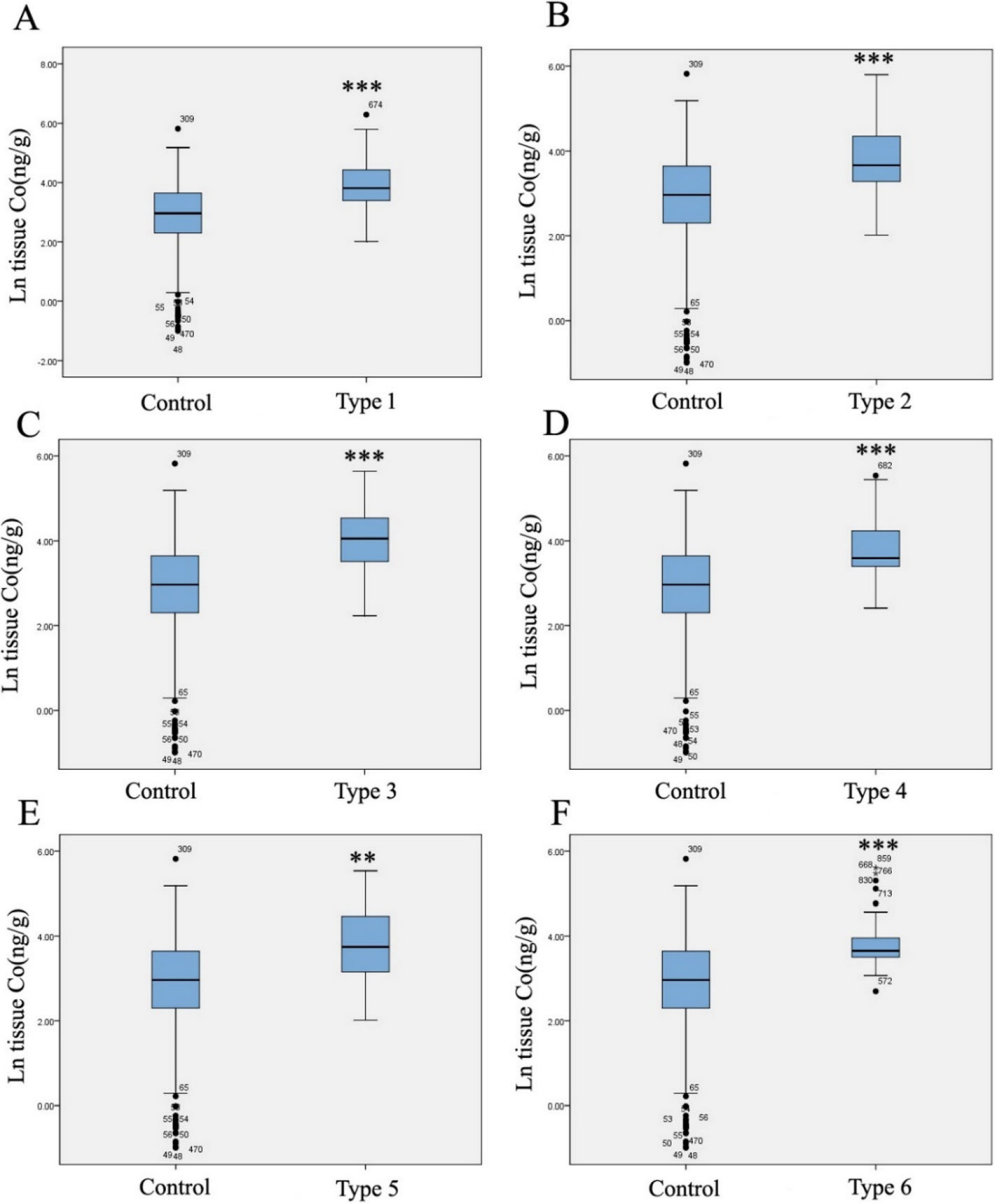
Levels of cobalt in CHD subtypes and control groups in fetus placental tissues. Boxplots of cobalt levels (Napierian logarithm transformed) of fetus placental tissue samples. The line inside the box = medians; the box length = interquartile range (IQR); the upper and lower ends = 95 and 5% value. One-sample Kolmogorov-Smirnov test was used to verify the distributions of cobalt. **P < 0.01 or ***P < 0.001, cases compared to the control group. Type1, septal defect; type 2, conotruncal defects; type 3, right ventricular outflow tract obstruction; type 4, left ventricular outflow tract obstruction; type 5, anomalous pulmonary venous return; type 6, other heart defects

The median (5–95% range) fetal placental cobalt concentrations were 19.350 ng/g (0.617 to 97.834 ng/g) and 42.500 ng/g (14.431 to 241.959 ng/g) in the control and case groups, respectively, which indicated that the concentration of fetal placental cobalt in the case group was significantly higher than that in the control group (*P* < 0.001). Fetal placental cobalt levels in each CHD subtype were significantly higher than those in controls (*P* < 0.01) (Table 3). As shown in Fig. 1 c and d, the distribution of cobalt in placental tissues was not normal. The data indicated that the concentrations of fetal placental tissue cobalt in the CHD group were significantly higher than that in the control group (*P* < 0.001) (Figs. 1 and 3).

**Table 3:** Descriptive statistics for placental tissue cobalt level in the case and control groups

	Tissue cobalt (ng/g)							
	*n*	AM	5th p	25th p	Median	75th p	95th p	*P* value
Control	212	30.705	0.617	9.965	19.350	38.233	97.834	
All cases	181	69.701	14.431	28.465	42.500	81.175	241.959	< 0.001***
Case with septal defects	124	71.953	13.550	29.660	45.230	85.458	239.226	< 0.001***
Case with conotruncal defects	96	65.971	12.883	26.450	38.960	77.843	244.043	< 0.001***
Case with right ventricular outflow tract obstruction	65	69.966	16.378	33.270	57.540	94.035	190.738	< 0.001***
Case with left ventricular outflow tract obstruction	29	61.630	12.880	29.575	36.260	73.260	241.515	0.001***
Case with anomalous pulmonary venous return	26	66.883	7.560	22.903	42.330	89.230	245.033	0.003**
Other heart defects	32	64.142	19.143	32.940	38.585	54.775	250.074	< 0.001***

*n* number; *AM* arithmetic means; *5th p*, *95th p* lead level in 5%, 95% percentiles respectively

*P < 0.05 or **P < 0.01, two-tailed test, Wilcoxon-Mann-Whitney on nonparametric test compared to the control group

### Maternal hair cobalt exposure and CHD risk

**Fig. 4:**
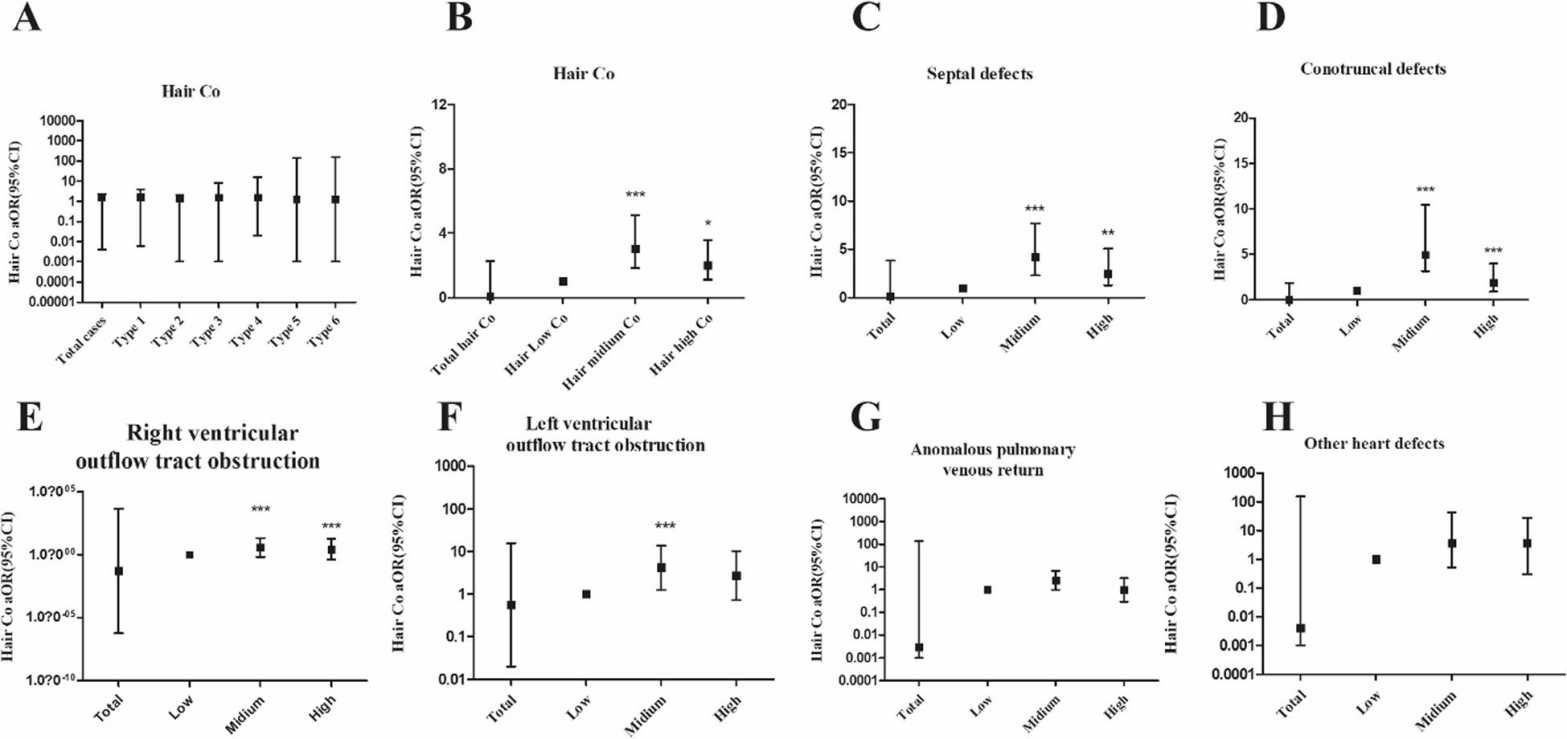
Association between mother hair cobalt levels and the risk of CHDs in offspring. The OR adjusted for maternal age, gestational age, education, taking folic acid (yes, no), parental smoking (yes, no), maternal pre-pregnancy BMI and iron, and calcium concentration. *P < 0.05, **P < 0.01, or ***P < 0.001. Type1, septal defect; type 2, conotruncal defects; type 3, right ventricular outflow tract obstruction; type 4, left ventricular outflow tract obstruction; type 5, anomalous pulmonary venous return; type 6, other heart defects

Logistic regression analysis showed that the overall risk of CHDs increases with hair cobalt concentrations (total CHDs aOR, 0.090; 95% CI, 0.004–2.264) after adjustment for potential risk factors. As shown in Table 4 and Fig. 4, significant differences were found in the middle and high concentrations of cobalt in total CHDs (middle cobalt level group, aOR, 3.035, 95% CI, 1.809–5.092, *P* < 0.001; high cobalt level group, aOR, 1.995, 95% CI, 1.116–3.569, *P* < 0.05). Similar results were found in the septal defect group (middle cobalt level group, aOR, 4.183; 95% CI 2.283–7.664; *P* < 0.001; high cobalt level group, aOR, 2.544; 95% CI, 1.289–5.021; *P* < 0.01), conotruncal defects (middle cobalt level group, aOR, 3.133; 95% CI 1.628–6.029; *P* < 0.001), right ventricular outflow tract obstruction (aOR, 3.657; 95% CI 1.805–7.408; *P* < 0.001; high cobalt level group, aOR, 2.717; 95% CI, 1.251–5.902; *P* < 0.05), and left ventricular outflow tract obstruction (middle cobalt level group, aOR, 4.168; 95% CI 1.254–13.854; *P* < 0.05) (Table 4 and Fig. 4). All the results indicated that hair cobalt exposure may increase the risk of CHDs in offspring.

**Table 4:** Risks for fetal CHD in different maternal hair cobalt concentrations

Group	Total hair Co	Hair low *n* = 197 (< 0.0161 ng/mg)	Hair medium *n* = 199 (0.0161 ng/mL–0.0435 ng/mg)	Hair high *n* = 197 (> 0.0435 ng/mg)
Control (*n*)	325	153	87	85
All cases (*n*)	268	44	112	112
cOR	1.761***	Reference	4.476***	4.582***
aOR	0.090	Reference	3.035***	1.995*
95% CI	0.004–2.264		1.809–5.092	1.116–3.569
Case with septal defects (*n*)	182	27	78	77
cOR	1.652	Reference	5.080***	5.133***
aOR	0.148	Reference	4.183***	2.544**
95% CI	0.006–3.815		2.283–7.664	1.289–5.021
Case with conotruncal defects (*n*)	133	21	59	53
cOR	1.528	Reference	4.941***	4.543***
aOR	0.001	Reference	3.133***	1.885
95% CI	0.001–1.865		1.628–6.029	0.899–3.952
Case with right ventricular outflow tract obstruction (*n*)	123	16	54	53
cOR	1.627**	Reference	5.935***	5.962***
aOR	0.016	Reference	3.657***	2.717***
95% CI	0.001–7.969		1.805–7.408	1.251–5.902
Case with left ventricular outflow tract obstruction (*n*)	41	5	20	16
cOR	1.679**	Reference	7.034***	5.760**
aOR	0.553	Reference	4.168***	2.701
95% CI	0.020–15.498		1.254–13.854	0.719–10.150
Case with anomalous pulmonary venous return (*n*)	42	8	19	15
cOR	1.483*	Reference	4.177**	3.375**
aOR	0.003	Reference	2.585	0.969
95% CI	0.001–138.136		0.991–6.746	0.296–3.174
Case with other heart defects (*n*)	48	10	18	20
cOR	1.521**	Reference	3.166**	3.600**
aOR	0.004	Reference	1.330	1.255
95% CI	0.001–156.466		0.500–3.536	0.424–3.710

*n* number, *aOR* adjusted odds ratio, *cOR* crude odds ratio

Logistic regression was used to calculate odds ratios and 95% CIs; the low-medium-high concentration of cobalt are referring to the tertiles and lose-dose group of cobalt was consider as a reference; all models were adjusted for maternal age, gestational age, education, taking folic acid (yes, no), parental smoking (yes, no), maternal pre-pregnancy BMI and iron, and calcium concentration. Significant differences between the mothers of case and control were indicated by the following: *P < 0.05, **P < 0.01 or ***P < 0.001

### Fetal placental tissue cobalt exposure and CHD risk

**Fig. 5:**
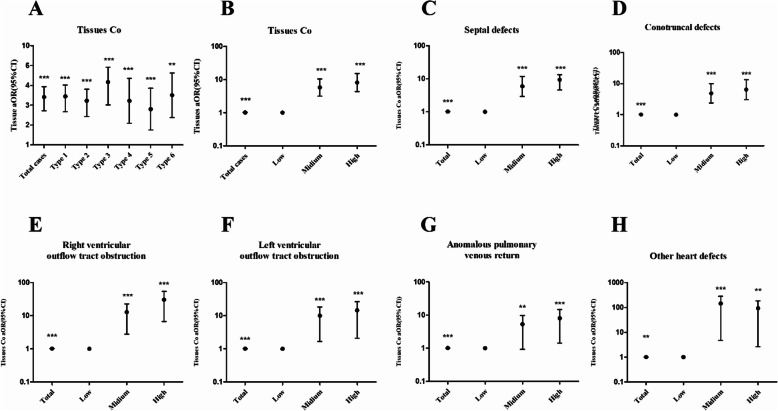
Association between fetus placental tissue cobalt levels and the risk of CHDs in offspring. The OR adjusted for maternal age, gestational age, education, taking folic acid (yes, no), parental smoking (yes, no), maternal pre-pregnancy BMI and iron, and calcium concentration. *P < 0.05, **P < 0.01, or ***P < 0.001. Type1, septal defect; type 2, conotruncal defects; type 3, right ventricular outflow tract obstruction; type 4, left ventricular outflow tract obstruction; type 5, anomalous pulmonary venous return; type 6, other heart defects. A, aOR of total tissues Co and 6 types of CHDs

As shown in Table 5, the cobalt level in fetal placental tissue increased the risk of CHDs in offspring (aOR 1.017, 95% CI 1.010–1.024, *P* < 0.001). The risk of each subtype of CHD also increased, including septal defects (aOR, 1.017; 95% CI, 1.010–1.024; *P* < 0.001), conotruncal defects (aOR, 1.015; 95% CI, 1.007–1.022; *P* < 0.001), right ventricular outflow tract obstruction (aOR, 1.022; 95% CI, 1.013–1.031; *P* < 0.001), left ventricular outflow tract obstruction (aOR, 1.012, 95% CI, 1.003–1.021; *P* < 0.05), anomalous pulmonary venous return (aOR, 1.013, 95% CI, 1.004–1.023; *P* < 0.01), and other heart defects (aOR, 1.013, 95% CI, 1.004–1.022; *P* < 0.001) compared with controls. The risk of CHDs and different levels of cobalt in fetal placental tissues was analyzed by trisecting the concentrations of all subjects. Table 5 and Fig. 5 show that exposure to the medium and highest fetal placental concentrations was associated with increased risks of all subtypes of CHDs (*P* < 0.01), except for anomalous pulmonary venous return subtype. The results suggested that the association between the cobalt level and CHD risk may also display a dose-response relationship in all the subtypes except for the other heart defects.

**Table 5:** Risks for fetal CHD subtypes in different fetal placental tissue cobalt levels

Group	Total tissue Ni	Tissue low, *n* = 131 (< 39.853 ng/g)	Tissue medium, *n* = 131 (39.853–75.894 ng/g)	Tissue high, *n* = 131 (> 75.894 ng/g)
Control (*n* )	212	109	58	45
All cases (*n* )	181	22	73	86
cOR	2.850***	Reference	6.236***	9.469***
aOR	1.017***	Reference	5.777***	8.162***
95% CI	1.010–1.024		3.185–10.480	4.379–15.212
Case with septal defects (*n* )	124	14	46	64
cOR	2.895***	Reference	6.175***	11.073***
aOR	1.017***	Reference	5.846***	9.230***
95% CI	1.010–1.024		2.904–11.769	4.519–13.347
Case with conotruncal defects (*n* )	96	14	38	44
cOR	2.568***	Reference	5.101***	7.613***
aOR	1.015***	Reference	4.853***	6.354***
95% CI	1.007–1.022		2.380–9.896	3.025–13.347
Case with right ventricular outflow tract obstruction (*n* )	65	5	21	39
cOR	3.610***	Reference	7.893***	18.893***
aOR	1.022***	Reference	8.014***	19.164***
95% CI	1.013–1.031		2.800–22.933	6.723–54.632
Case with left ventricular outflow tract obstruction (*n* )	29	4	12	13
cOR	2.454***	Reference	5.638**	7.872**
aOR	1.012***	Reference	5.551***	7.550***
95% CI	1.003–1.021		1.669–18.465	2.112–26.995
Case with anomalous pulmonary venous return (*n* )	26	5	9	12
cOR	2.301***	Reference	3.383*	5.813**
aOR	1.013***	Reference	3.023**	4.589***
95% CI	1.004–1.023		0.938–9.740	1.436–14.664
Case with other heart defects (*n* )	32	1	21	10
cOR	2.695***	Reference	39.466***	24.222**
aOR	1.013**	Reference	36.664***	22.082**
95% CI	1.004–1.022		4.727–284.371	2.636–184.948

*n* number, *aOR* adjusted odds ratio, *cOR* crude odds ratio

Logistic regression was used to calculate odds ratios and 95% CIs; the low-medium-high concentration of cobalt are referring to the tertiles and lose-dose group of cobalt was consider as a reference; all models were adjusted for maternal age, gestational age, education, taking folic acid (yes, no), parental smoking (yes, no), maternal pre-pregnancy BMI and iron, and calcium concentration. Significant differences between the mothers of case and control were indicated by the following: *P < 0.05, ***P < 0.001 or **P < 0.01

## Discussion

The study showed that the levels of cobalt in maternal hair samples and fetal placental tissues were both higher in the CHD groups than levels in the control groups (Table 2 and Table 3). Logistic analysis indicated that maternal exposure to cobalt had a significant association with the risk of CHDs in offspring, and higher concentrations of cobalt may increase the risk of CHDs in some subtypes in offspring (Tables 4 and 5 and Figs. 4 and 5).

CHDs were caused by inherited factors, environmental exposure, or both [33, 34]. Metal exposure was one of the pathogenic factors [35]. Other metals, such as lead, arsenic, and barium, were associated with the occurrence of CHDs in offspring [12, 35]. Placental chromium, manganese, and lead have been reported to be toxic to children [36]. Cobalt ions were more bioavailable to interact with various cellular receptors, biomolecules, and ion channels [18, 22]. In the study, logistic analysis was adjusted for iron concentration and calcium concentration that was because iron could significantly inhibit the absorption of cobalt in both dietary cobalt treatments [37]. Besides, cobalt causes contraction of vascular smooth muscle depending activation of calcium channels in the plasma membrane [38]. Although cobalt had many physiological implications, cobalt toxicity had been shown in heart, liver, lung, and the neurological system [27, 39]. Our study provided evidence for the cardiac toxicity of chronic cobalt exposure in pregnant women. Pregnant women might have little acute occupational exposure to cobalt, but avoiding environmental exposure was more difficult to anticipate, measure, or control.

In order to observe the long-term cobalt exposure of pregnant mothers, hair samples were utilized. Hair samples may even provide us with information regarding the metal exposure of the mother prior to pregnancy. In addition, cobalt in fetal placental tissues was also analyzed in the study. Cobalt levels in fetal placental tissues could directly display cobalt exposure during pregnancy. Besides, the samples were recruited in multiple hospitals in different provinces of China, and the sample size of pregnant women was superior in previous studies, which could reflect the exposure of cobalt in different areas in China during pregnancy.

Our study had many limitations. First, the mechanism of cobalt exposure-induced CHDs has not been shown in this study, and further studies are warranted to clarify the underlying mechanisms. The possible mechanisms of the toxic effect of cobalt were that cobalt could attack fatty acids and convert them to free radical lipids and free radical lipid peroxidation, leading to damage to the cell membrane fluidity and interruption of reactive oxygen species [40]. Cobalt ions could occupy the combining site of calcium and ions and inhibit the activity of Ca^2+^ ATPase, which may impair heart development [18]. Second, this study did not consider interactions between cobalt and all the other metals except calcium and ions. This was because cobalt (Co^2+^) uptake appears to be shared with calcium [22], and cobalt gastrointestinal absorption involves mechanisms with ions [18]. This is an area that should be further investigated in future studies. Third, other samples, such as urine and serum, should be studied in the future. Fourth, our study showed that cobalt exposure was related to CHD occurrence in offspring. However, the range of the 95% CI was wide, perhaps because of the small number of CHDs. Further studies are needed to recruit more CHD cases in the future.

## Conclusion

We observed an association between the cobalt concentrations in maternal and fetal placental tissues and the increased risk of CHDs in the general pregnant population with exposure to cobalt.

## Abbreviations

CHD: Congenital heart disease
aOR: Adjusted odds ratio
cOR: Crude odds ratio
95% CI: 95% confidence interval
GM: Geometric mean
BMI: Body mass index
ICP-MS: Inductively coupled plasma mass spectrometry
